# Supplementary material for: Comparative Proteomic Analysis of Protein Patterns of Stenotrophomonas maltophilia in Biofilm and Planktonic Lifestyles
Source: Microorganisms. 2023 Feb 9;11(2):442. doi: 10.3390/microorganisms11020442 (PMC9960084; doi:10.3390/microorganisms11020442)
Supplement: Supplementary file 1 [file microorganisms-11-00442-s001.zip › microorganisms-2168477-supplementary.pdf]

Table S1. Proteins hyper-expressed (n = 42; ≥ 1.5-fold *vs*. planktonic cells; $p < 0.05$, *t*-test) in *S. maltophilia* Sm126 biofilm cells. Molecular weight (MW), isoelectric point (pI), and biofilm/planktonic ratio are shown.

Spot ID	MW (dalton)	pI	Ratio (fold)
**SSP 0106**	26,178	4.61	2.9
**SSP 0204**	29,694	4.50	5.8
**SSP 0306**	29,992	4.55	2.7
**SSP 1202**	31,495	4.75	2.3
**SSP 1303**	36,202	4.71	1.9
**SSP 1313**	32,299	4.73	2.7
**SSP 1504**	42,779	4.74	1.7
**SSP 1505**	44,137	4.69	6.2
**SSP 1507**	43,861	4.79	3.7
**SSP 1806**	72,144	4.79	3.0
**SSP 2501**	49,234	4.99	1.6
**SSP 2508**	44,878	5.51	1.6
**SSP 3405**	35,776	5.58	2.6
**SSP 4713**	69,065	6.06	7.8
**SSP 4806**	85,923	5.99	9.5
**SSP 4807**	82,630	6.05	10.9
**SSP 5108**	20,376	6.58	2.5
**SSP 5306**	27,352	6.66	3.9
**SSP 6308**	33,491	7.14	1.6
**SSP 6403**	43,319	6.91	4.7
**SSP 6405**	41,493	6.93	4.3
**SSP 6407**	35,468	6.99	2.2
**SSP 6411**	38,322	7.07	1.9
**SSP 7002**	10,913	7.16	5.7
**SSP 7003**	18,566	7.20	4.1
**SSP 7101**	24,854	7.22	3.4
**SSP 7303**	35,401	7.26	3.5
**SSP 7304**	35,942	7.42	1.9
**SSP 7403**	44,035	7.53	2.2

**SSP 7502**	49,476	7.44	10.1
**SSP 7601**	55,676	7.70	1.7
**SSP 8201**	29,703	7.93	2.7
**SSP 8203**	26,876	8.03	1.6
**SSP 8210**	24,600	8.43	1.7
**SSP 8401**	41,227	7.97	2.2
**SSP 8402**	40,705	8.08	2.3
**SSP 8406**	42,730	8.56	7.8
**SSP 8501**	46,176	7.97	3.5
**SSP 8602**	60,821	7.97	5.2
**SSP 8605**	58,479	8.18	2.0
**SSP 8706**	69,875	8.10	3.9
**SSP 8813**	81,617	8.95	2.9

Table S2. Proteins (n=34) expressed exclusively by *S. maltophilia* Sm126 biofilm cells. Molecular weight (MW) and isoelectric point (pI) are shown.

Spot ID	MW (dalton)	pI
**SSP 0107**	12,567	4.62
**SSP 1305**	36,096	4.83
**SSP 3001**	6,924	5.53
**SSP 3102**	20,525	5.56
**SSP 3207**	24,355	5.83
**SSP 3712**	67,921	5.61
**SSP 3808**	79,698	5.63
**SSP 4409**	39,353	6.17
**SSP 5613**	52,720	6.38
**SSP 5616**	56,576	6.54
**SSP 5617**	54,309	6.58
**SSP 5714**	61,410	6.58
**SSP 6109**	17,758	6.61
**SSP 6314**	27,123	6.93
**SSP 6607**	59,897	6.75
**SSP 6708**	72,075	6.60
**SSP 7007**	11,577	7.19
**SSP 7203**	26,920	7.24
**SSP 7208**	26,409	7.86
**SSP 7401**	44,007	7.35
**SSP 7501**	49,983	7.19
**SSP 7503**	44,842	7.77
**SSP 7703**	72,253	7.58
**SSP 8011**	9,775	8.17
**SSP 8013**	8,313	8.50
**SSP 8205**	27,922	8.53
**SSP 8206**	29,196	8.76
**SSP 8407**	40,191	8.61
**SSP 8408**	42,122	9.00
**SSP 8509**	45,239	8.66

**SSP 8511**	45,508	8.53
**SSP 8812**	83,683	8.73
**SSP 9202**	26,817	9.39
**SSP 9301**	30774	8.96

Table S3. Proteins hyper-expressed (n = 56; ≥ 1.5-fold *vs*. biofilm cells; $p<0.05$, t-test) in *S. maltophilia* Sm126 planktonic cells. Molecular weight (MW), isoelectric point (pI), and planktonic/biofilm ratio are shown.

Spot ID	MW (dalton)	pI	Ratio (fold)
**SSP 0009**	10,310	4.17	5.5
**SSP 0011**	10,677	4.51	4.0
**SSP 0108**	12,940	4.30	2.8
**SSP 0111**	14,494	4.42	3.6
**SSP 0201**	31,825	4.34	4.4
**SSP 0208**	25,141	4.53	3.5
**SSP 0304**	37,464	4.36	19.0
**SSP 0305**	40,151	4.37	10.9
**SSP 0403**	41,681	4.43	2.2
**SSP 0701**	66,049	4.37	12.1
**SSP 0802**	78,629	4.33	7.8
**SSP 1006**	8,238	4.90	10.3
**SSP 1101**	26,333	4.73	4.8
**SSP 1103**	20,847	4.95	2.3
**SSP 1105**	14,210	4.64	4.4
**SSP 1106**	14,082	4.74	6.5
**SSP 1206**	24,302	4.73	2.1
**SSP 1302**	35,245	4.70	3.7
**SSP 2002**	8,072	5.42	4.2
**SSP 2005**	12,795	5.31	2.9
**SSP 2006**	9,398	5.04	9.4
**SSP 2102**	26,147	5.15	5.0
**SSP 2104**	16,992	5.31	4.4
**SSP 2203**	30,126	5.13	3.0
**SSP 2204**	26,972	5.14	2.0
**SSP 2207**	25,895	5.36	5.2
**SSP 2407**	35,064	5.17	1.5
**SSP 2701**	63,080	4.99	4.1
**SSP 3206**	25,622	5.54	3.5

**SSP 4106**	16,406	5.97	13.5
**SSP 4202**	25,203	6.00	2.1
**SSP 5001**	13,212	6.13	4.4
**SSP 5008**	11,734	6.12	1.6
**SSP 5504**	40,648	6.31	5.6
**SSP 6004**	9,137	6.60	6.9
**SSP 6101**	22,111	6.61	3.9
**SSP 6110**	13,839	7.11	1.8
**SSP 6410**	40,426	7.12	2.7
**SSP 6714**	63,839	7.11	4.0
**SSP 7004**	15,867	7.88	8.5
**SSP 7005**	8,269	7.50	3.1
**SSP 7106**	18,601	7.34	7.5
**SSP 7201**	29,808	7.21	4.0
**SSP 7204**	28,058	7.30	1.7
**SSP 7404**	37,846	7.53	3.1
**SSP 8002**	10,127	8.17	5.5
**SSP 8004**	7,464	8.80	9.8
**SSP 8006**	8,429	9.09	2.2
**SSP 8009**	7,237	8.21	3.6
**SSP 8207**	23,486	9.07	2.7
**SSP 8209**	25,480	8.83	2.6
**SSP 8212**	26,548	8.58	3.4
**SSP 8214**	27,220	9.19	2.1
**SSP 8512**	44,280	8.44	7.2
**SSP 9003**	19,525	9.44	2.7
**SSP 9102**	23,200	9.50	1.5

Table S4. Proteins (n=17) expressed exclusively by *S. maltophilia* Sm126 planktonic cells. Molecular weight (MW) and isoelectric point (pI) are shown.

Spot ID	MW (dalton)	pI
**SSP 0109**	17,906	3.96
**SSP 0110**	20,203	3.95
**SSP 0209**	24,635	4.21
**SSP 0308**	31,083	4.51
**SSP 2007**	10,290	5.31
**SSP 2008**	10,767	5.45
**SSP 3007**	10,333	5.64
**SSP 4107**	20,144	5.85
**SSP 5405**	37,236	6.52
**SSP 5407**	37,060	6.27
**SSP 5611**	47,507	6.33
**SSP 6008**	9,423	6.83
**SSP 6009**	8,198	6.71
**SSP 6414**	35,714	6.85
**SSP 8109**	19,583	8.49
**SSP 8110**	18,553	8.22
**SSP 9104**	14,418	10.00

8812

509
